# Sex differences in health-related quality of life after renal cell carcinoma surgery: a population-based study in Sweden

**DOI:** 10.1007/s11136-025-04157-w

**Published:** 2026-01-14

**Authors:** Stephanie E. Bonn, Bodil Westman, Maria E. C. Schelin, Christel Hedman, Börje Ljungberg, Andreas Karlsson Rosenblad

**Affiliations:** 1 Department of Medicine Solna, Division of Clinical Epidemiology, Karolinska Institutet, Stockholm, Sweden; 2 Regional Cancer Centre Stockholm-Gotland, Region Stockholm, Stockholm, Sweden; 3 Department of Care Science, Sophiahemmet University, Stockholm, Sweden; 4 Institute for Palliative Care, Region Skåne, Department of Clinical Sciences Lund, Lund University, Lund, Sweden; 5 Department of Molecular Medicine and Surgery, Karolinska Institutet, Stockholm, Sweden; 6 R & D Department, Stockholms Sjukhem Foundation, Stockholm, Sweden; 7 Department of Diagnostics and Intervention, Urology and Andrology, Umeå University, Umeå, Sweden; 8 Department of Statistics, Uppsala University, Uppsala, Sweden; 9 Department of Medical Sciences, Division of Clinical Diabetology and Metabolism, Uppsala University, Uppsala, Sweden; 10 Department of Neurobiology, Care Sciences and Society, Division of Family Medicine and Primary Care, Karolinska Institutet, Stockholm, Sweden

**Keywords:** Cohort study, Health-related quality of life, Kidney cancer, Renal cell carcinoma, Sex differences, Surgery

## Abstract

**Purpose:**

To examine sex differences in health-related quality of life (HRQoL) among patients surgically treated for renal cell carcinoma (RCC) in Sweden, utilizing data from the National Swedish Kidney Cancer Register (NSKCR).

**Methods:**

In this study of 4658 surgically treated RCC patients, data on HRQoL, clinical, demographic, and socioeconomic characteristics were retrieved from the NSKCR for patients undergoing surgical treatment between January 2016, and April 2024. HRQoL was measured using the 14- and 19-item versions of the Functional Assessment of Cancer Therapy – Kidney Symptom Index (FKSI-14/19) instrument six months after surgery. The association between sex and HRQoL was estimated using linear regression. Separate analyses were performed for the FKSI-14 and FKSI-19 total scores and underlying domains.

**Results:**

In total, 3086 (66.3%) men and 1572 (33.7%) women were included. After adjusting for clinical, demographic, and socioeconomic characteristics, male sex was significantly associated with higher HRQoL. Specifically, men had higher scores, indicating fewer symptoms, for physical and mental symptoms according to FKSI-14 (*P* < 0.001), and for physical (*P* < 0.001) and emotional (*P* < 0.001) disease-related symptoms, as well as treatment side effects (*P* < 0.022), according to FKSI-19. Total HRQoL was significantly higher in men, according to both the FKSI-14 (*P* < 0.001) and the FKSI-19 (*P* < 0.001).

**Conclusions:**

HRQoL differed significantly between men and women six months after surgery, with men reporting higher HRQoL, even after accounting for clinical, demographic, and socioeconomic factors. Healthcare professionals should be aware of the risk of lower HRQoL among female patients.

## Abstract (plain language summary)

Survival after a kidney cancer diagnosis has improved significantly in recent decades, meaning that the number of survivors is growing. To provide better support for patients’ well-being, it is important to understand what influences the quality of life after treatment. In this study, we investigated whether there are differences in health-related quality of life between men and women who have undergone surgery for renal cell carcinoma, the most common type of kidney cancer. We analysed data from over 4500 patients in Sweden, about one-third of whom were women. Our findings showed that men reported better health-related quality of life than women, even after considering differences in for example age, health and socioeconomic factors. Our results suggest that more personalized support may be needed, especially for women, to reduce the unjustified differences in health-related quality of life between women and men.

## Introduction

More than 430,000 individuals are diagnosed with kidney cancer worldwide every year, corresponding to 2.2% of all cancers [1]. The incidence is increasing in European countries and in younger populations [2]. In Sweden, approximately 1200 individuals are diagnosed yearly [3]. Renal cell carcinoma (RCC) accounts for > 90% of all kidney cancers [4].

Survival rates after RCC depend heavily on tumour stage but are high in patients without metastasis at the time of diagnosis. Most patients with localized RCC undergo surgical treatment [5]. Similar to observed trends in increasing survival of solid tumours [6], the overall survival rates in patients with RCC in Sweden have increased dramatically during the past decades, from a 5 year survival rate of around 30% in the 1960s to over 80% today [3].

As a result, the accumulated number of people living with a kidney cancer diagnosis has increased rapidly, making it increasingly important to address patient-reported outcomes. Patient-reported outcome measures (PROMs) are standardized instruments that capture patients’ own perceptions of their symptoms, functioning, and well-being [7]. One key outcome measured using PROMs is health-related quality of life (HRQoL), a multidimensional construct that reflects the impact of disease and treatment on e.g. physical, psychological, and social functioning [8]. To better identify patients who would benefit from additional support, a deeper understanding of the factors influencing HRQoL is needed.

A systematic review by O’Dea et al. [9] highlighted that people diagnosed with kidney cancer experience a wide range of unmet supportive care needs, particularly related to psychological and physical well-being. Moreover, high levels of distress and depressive symptoms, psychological components that may influence HRQoL, have been reported [10]. Despite the favourable prognosis, patients with localized RCC report high levels of anxiety and distress related to fear of recurrence [11, 12], with distress persisting for a long time [12, 13], which can negatively affect HRQoL.

While some studies have shown reduced patient-reported quality of life [14, 15], others report no long-term differences between RCC patients and the general population [11, 16, 17]. Female RCC patients have been reported to experience higher levels of psychosocial and physical distress than male patients after diagnosis, biopsy, and surgery [18], as well as lower HRQoL [19]. However, the underlying reasons for these sex differences in HRQoL remain unclear. It is not clarified whether they are attributable to clinical, demographic, or socioeconomic factors.

Previous studies addressing sex differences in HRQoL after RCC have been limited in sample size, calling for further research using larger samples. The National Swedish Kidney Cancer Register (NSKCR) [3], a national quality register aimed at measuring and improving the quality of care for Swedish RCC patients, has collected data on HRQoL from more than 4600 surgically treated patients using the Functional Assessment of Cancer Therapy – Kidney Symptom Index (FKSI-19) instrument, a validated PROM for this patient population [20, 21]. In combination with data on clinical, demographic, and socioeconomic factors from the register, this provides a unique resource for investigating HRQoL in a large, representative patient population.

### Aim

The aim of the present population-based, real-world cohort study was to examine sex differences in HRQoL six months after surgery among RCC patients in Sweden, using data from the NSKCR and considering differences in clinical, demographic, and socioeconomic factors.

### Methods and material

The NSKCR has collected data on diagnosis, treatment, and tumour characteristics for Swedish RCC patients since 2005. Although participation in NSKCR is voluntary, it has an almost complete coverage when compared with the National Swedish Cancer Register, to which healthcare providers are obliged by law to provide information about all cancer cases [22, 23]. PROM data, including HRQoL, measured six months after surgery have been collected for patients undergoing surgical treatment for RCC since January 2016. Further details about the NSKCR and the data collection process for PROM data may be found in previously published studies [22–26]. This study is reported in accordance with the *Strengthening the Reporting of Observational studies in Epidemiology* (STROBE) guidelines [27].

### Study population

**Fig. 1:**
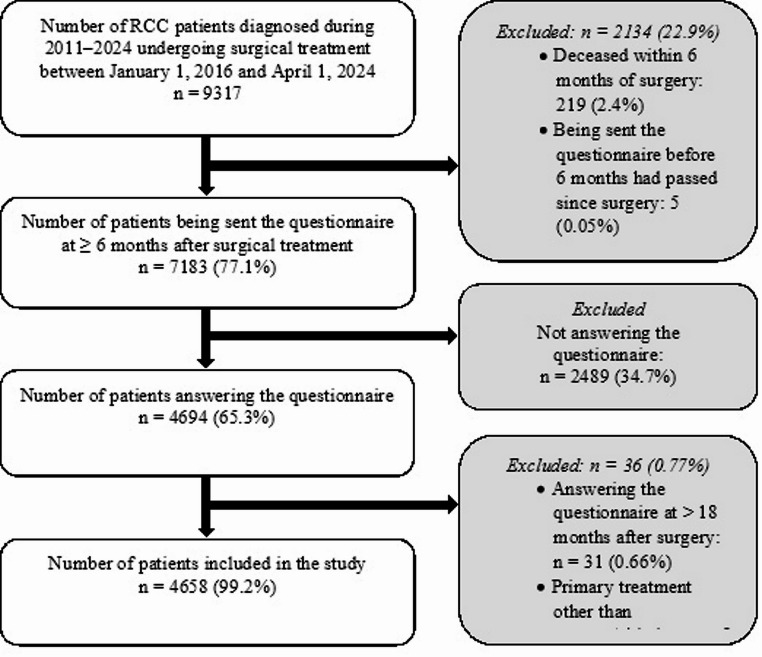
Flowchart of the inclusion process for the 4658 renal cell carcinoma (RCC) patients included in the present study

On October 1, 2024, data on clinical variables and answers to the PROM questionnaire were retrieved from the NSKCR for all 9317 patients diagnosed with RCC who had received surgical treatment between January 1, 2016, and April 1, 2024. We excluded patients who had died within six months of surgery (*n* = 219), had been sent the questionnaire before six months had passed since the surgery (*n* = 5), or for various reasons (e.g. administrative oversights and errors) had not been sent the questionnaire (*n* = 1910). Of the 7183 (77.1%) patients being sent the questionnaire at ≥ 6 months after surgical treatment, a total of 4694 (65.3%) answered the questionnaire. We further excluded 36 individuals who had either answered the questionnaire at > 18 months after surgery (*n* = 31) or had a primary treatment other than surgery/ablation registered in the NSKCR (*n* = 5). The final study population comprised 4658 patients. An overview of the inclusion process is given in Fig. 1.

### Assessment of health-related quality of life

In the NSKCR, HRQoL is measured using the validated 19-item version of the Functional Assessment of Cancer Therapy – Kidney Symptom Index (FKSI-19) instrument [20, 21]. The FKSI was originally developed to provide a comprehensive measure of disease and treatment related symptoms among patients with advanced kidney cancer [28]. Answers are given on a five-level Likert scale (“Not at all”, “A little bit”, “Somewhat”, “Quite a bit”, or “Very much”), with five items scored 0 to 4 and the remaining 14 items reverse-scored 4 to 0, resulting in a total score range of 0–76 points. The FKSI-19 is divided into four domains: Disease-related symptoms – Physical (DRS-P; 12 items, score range 0–48 points), Disease-related symptoms – Emotional (DRS-E; 1 item, score range 0–4 points), Treatment side effects (TSE; 3 items, score range 0–12 points), and Function/Well-being (FWB; 3 items, score range 0–12 points).

While the FKSI-19 is an established instrument, allowing comparability with previous studies, we also addressed HRQoL using a trimmed, 14-item version of questionnaire, based on the results from a previous psychometric evaluation of the 19-item version in the NSKCR [23]. This evaluation showed that the four-factor structure of the 19-item version provided only a barely acceptable model fit, suggesting that an alternative 14-item trimmed version (FKSI-14) with a two-factor structure may better reflect the underlying symptom experience while being less burdensome for patients to answer. FKSI-14 has a total score range of 0–56 points and is divided into the two domains Physical and mental symptoms (PMS; 9 items, 0–45 points) and Daily life function and well-being (DLFWB; 5 items, 0–20 points). For both the FKSI-19 and FKSI-14, higher scores indicate fewer symptoms, i.e., a score of 0 indicates a severely symptomatic patient, while the maximum score indicates a completely asymptomatic patient.

### Study variables and data collection

Data on date of birth, sex, education level, marital status, current occupation, body mass index (BMI; kg/m^2^) at the time of surgery, date of RCC diagnosis, date of surgery, type of surgery, date of answering the FKSI-19 questions, currently on treatment for RCC, comorbidities, tumour size (mm), Tumour-Node-Metastasis (TNM) stage, RCC type, and FKSI-19 score were retrieved from the NSKCR. Date of birth and sex were obtained from each individual’s Swedish Personal Identification Number. Education level, marital status, occupation, current treatment for RCC, comorbidities, and FKSI-19 data were collected using a register-specific questionnaire sent to the surgically treated patients six months after surgery. The questionnaires could be answered either on paper or online. All remaining variables were reported to the NSKCR from the hospital or clinic treating the patient.

Age (years) at surgery was calculated from date of birth to date of surgery. Sex was categorized as male sex (yes/no). Education level was classified into three categories: compulsory school, upper-secondary school, and college/university. Marital status was categorized as married/cohabiting/partner (yes/no), where “yes” included individuals who were married/cohabiting or had a partner. For current occupation, respondents could select multiple prespecified alternatives. In this study, the alternatives employed, on sick leave, and retired were used as separate dichotomous variables, with those selecting the alternative in question being classified as “yes” and all the others being classified as “no”. Time (days) from RCC diagnosis to surgery was calculated from date of diagnosis to date of surgery. Time from surgery to answering the questionnaire was calculated as the number of days between surgery and the start date of online responses, or, if answered on paper, the date the responses were entered into the database.

The variable currently on treatment for RCC (yes/no) was self-reported and measured using the single question “Do you currently have any treatment for kidney cancer?”. Comorbidities were likewise self-reported and participants were asked whether they had any other permanent illness or disability diagnosed by a physician. The questionnaire listed ten prespecified diagnosis groups, with examples worded as follows: Another cancer, Physical handicap/disability, Disease of the muscles or bones (e.g. rheumatoid arthritis), Disease of the circulatory system (e.g. high blood pressure, cardiovascular disease), Disease of the respiratory system (e.g. asthma), Mental illness/emotional problems, Metabolic disease (e.g. diabetes, goitre), Neurological disease or disease of the sense organs (e.g. epilepsy, hearing loss), Skin disease/eczema/allergy, and Disease of the stomach/digestive organs (e.g. gastritis, gallstones, liver, or intestinal disease). A comorbidity index was constructed by giving each participant 1 point for each selected diagnosis group, resulting in an index score of 0–10 points.

In the NSKCR, tumour staging is performed according to the TNM 2017 classification system [29], with tumour size defined as the maximal tumour diameter obtained using computed tomography or magnetic resonance imaging, while histopathological classification is performed according to the 2016 World Health Organization (WHO) classification [30]. Based on the TNM stage, the two categorical variables metastatic RCC (yes/no) and T stage (T1a, T1b, T2, or T3–T4) were constructed. RCC type was categorized as: Clear cell, Papillary, Chromophobe, or Other RCC types. Type of surgery was classified as open or non-open (e.g. laparoscopy or robot-assisted laparoscopy) and dichotomized as open surgery (yes/no).

### Statistical analyses

Categorical data are presented as frequencies and percentages, n (%), while ordinal, discrete and continuous data are given as mean values with standard deviations (SDs), supplemented with medians and interquartile ranges (IQRs). Tests of differences between two groups were performed using Pearson’s χ^2^-test for categorical data, the Mann-Whitney test for ordinal and discrete data, and Welch’s *t*-test for continuous data. Given the large sample size and the reasonable many possible scale points for the FKSI-14/19 instruments, the FKSI-14/19 scores were considered being approximately continuous for the purpose of being used as outcomes in regression models. The magnitude of the association between sex and HRQoL was estimated using simple and multiple linear regression models separately for the FKSI-14/19 total scores and underlying domains, with male sex (yes/no) as the exposure of main interest. Besides sex, the multiple regression analyses included age at surgery, education level (reference category: compulsory school), married/cohabiting/partner, employed, on sick leave, retired, BMI, time from RCC diagnosis to surgery, time from surgery to answering the questionnaire, currently on treatment for RCC, comorbidity index, tumour size, metastatic RCC, open surgery, T stage (reference category: T1), and RCC type (reference category: clear cell) as independent variables. For dichotomous variables, “no” was used as reference category. The regression models were estimated using the HC3 version of heteroscedasticity-consistent covariance matrix estimation [31], with results reported as slope coefficient β with accompanying 95% confidence intervals (CIs). Missing values were in all analyses handled using pairwise deletion. All statistical analyses were performed using R 4.4.1+ (R Foundation for Statistical Computing, Vienna, Austria) with P-values < 0.05 considered statistically significant.

## Results

Clinical and demographic characteristics of the 4658 surgically treated RCC patients are given in Table 1. There were twice as many men as women, 3086 (66.3%) compared to 1572 (33.7%). The mean (SD) age at surgery was 67.2 (11.2) years, with most being between 50 and 79 years old (*n* = 3855; 82.8%). Only 371 (8.0%) participants were younger than 50 years. The mean (SD) BMI for the participants was 27.6 (5.7) kg/m^2^. Upper-secondary school was the most common education level, reported by 1726 (37.8%) of the participants, with 1448 (31.7%) having a college or university education. While no significant sex differences were observed for age, BMI, or education level, significantly more men than women were married, cohabiting, or had a partner (*P* < 0.001), 2407 (79.5%) compared to 1039 (67.2%). Likewise, significantly more men (*n* = 976; 31.6%) than women (*n* = 449; 28.6%) reported being employed (*P* = 0.035). In total, 270 (5.8%) of the participants were on sick leave while 2987 (64.1%) were retired, with no significant sex differences observed.

**Table 1:** Clinical and demographic characteristics of the 4658 surgically treated renal cell carcinoma (RCC) patients included in the present study

	All	Male	Female		Missing values, *n* (%)
Variable	*n* = 4658 (100%)	*n* = 3086 (66.3%)	*n* = 1572 (33.7%)	*P*-value	
*Age at date of surgery (years)*					
Mean (SD)	67.2 (11.2)	67.3 (11.0)	66.9 (11.4)	0.345^a^	0 (0.0)
Median (IQR)	69.2 (60.4–75.3)	69.3 (60.8–75.3)	69.0 (59.9–75.4)		
*Body Mass Index (kg/m* ^*2*^ *)*					
Mean (SD)	27.6 (5.7)	27.6 (5.8)	27.6 (5.5)	0.729^a^	94 (2.0)
Median (IQR)	26.9 (24.2–30.3)	27.1 (24.4–30.1)	26.7 (23.7–30.8)		
*Age groups, n (%)*				0.699^b^	0 (0.0)
< 50 years old	371 (8.0)	237 (7.7)	134 (8.5)		
50−69 years old	2104 (45.2)	1396 (45.2)	708 (45.0)		
70−79 years old	1751 (37.6)	1171 (37.9)	580 (36.9)		
≥ 80 years old	432 (9.3)	282 (9.1)	150 (9.5)		
*Education level, n (%)*				0.057^b^	90 (1.9)
Compulsory school	1394 (30.5)	922 (30.4)	472 (30.7)		
Upper-secondary school	1726 (37.8)	1179 (38.9)	547 (35.6)		
College/University	1448 (31.7)	931 (30.7)	517 (33.7)		
Married/Cohabiting/Partner, n (%)	3446 (75.4)	2407 (79.5)	1039 (67.2)	**< 0.001** ^b^	86 (1.8)
*Current occupation, n (%)* ^*d*^					
Employed	1425 (30.6)	976 (31.6)	449 (28.6)	**0.035** ^b^	0 (0.0)
On sick leave	270 (5.8)	173 (5.6)	97 (6.2)	0.476^b^	0 (0.0)
Retired	2987 (64.1)	1998 (64.7)	989 (62.9)	0.230^b^	0 (0.0)
*Time from RCC diagnosis to surgery (days)*					
Mean (SD)	97 (135)	96 (128)	100 (149)	0.322^a^	0 (0.0)
Median (IQR)	64 (39–110)	65 (40–111)	62 (38–107)		
*Time from surgery to answering the questionnaire (days)*					
Mean (SD)	250 (64)	249 (62)	252 (66)	0.121^a^	0 (0.0)
Median (IQR)	225 (211–262)	225 (211–260)	226 (211–265)		
Currently on treatment for RCC, n (%)	392 (8.7)	293 (9.7)	99 (6.5)	**< 0.001** ^b^	127 (2.7)
*Comorbidity index (0–10 points)*					
Mean (SD)	1.51 (1.48)	1.42 (1.42)	1.67 (1.58)	**< 0.001** ^c^	0 (0.0)
Median (IQR)	1 (0–2)	1 (0–2)	1 (1–2)		
*Tumour size (mm)*					
Mean (SD)	53.1 (34.6)	53.6 (35.4)	52.1 (32.8)	0.177^a^	2 (0.04)
Median (IQR)	43 (27–70)	43 (27–70)	43 (26.2–70)		
Metastatic RCC, n (%)	234 (5.0)	162 (5.3)	72 (4.6)	0.357^b^	1 (0.02)
Open surgery, n (%)	1770 (38.8)	1159 (38.3)	611 (39.6)	0.437^b^	91 (2.0)
T stage, n (%)				0.480^b^	42 (0.9)
T1a	2092 (45.3)	1375 (45.0)	717 (46.0)		
T1b	940 (20.4)	610 (20.0)	330 (21.2)		
T2	481 (10.4)	325 (10.6)	156 (10.0)		
T3–T4	1103 (23.9)	747 (24.4)	356 (22.8)		
*RCC type, n (%)*				**< 0.001** ^b^	4 (0.1)
Clear cell	3298 (70.9)	2159 (70.0)	1139 (72.5)		
Papillary	731 (15.7)	553 (17.9)	178 (11.3)		
Chromophobe	433 (9.3)	257 (8.3)	176 (11.2)		
Other	192 (4.1)	114 (3.7)	78 (5.0)		

IQR, interquartile range; SD, standard deviation. Significant *P*-values are given in **bold**. *P*-values from: ^a^Welch’s *t*-test; ^b^Pearson’s χ^2^-test; ^c^the Mann-Whitney test. ^d^Multiple choices possible

The mean (SD) time from receiving the RCC diagnosis until surgical treatment was 97 (135) days, while 250 (64) days had passed from the date of surgery until answering the questionnaire, with no significant sex differences. However, significantly more men (*n* = 293; 9.7%) than women (*n* = 99; 6.5%) reported in the questionnaire that they were receiving treatment for RCC (*P* < 0.001), while the comorbidity index was significantly lower for men than women, mean (SD) 1.42 (1.42) compared to 1.67 (1.58) points (*P* < 0.001).

The overall mean (SD) tumour size was 53.1 (34.6) mm. Notably, only 234 (5.0%) of the participants had metastatic RCC. Open surgery was performed on 1770 (38.8%) of the patients. T1a-stage was observed among 2092 (45.3%) patients, T1b in 940 (20.4%), and T3–T4 in 1103 (23.9%). Neither tumour size nor occurrence of metastatic RCC, use of open surgery, or distribution of T stage differed significantly between men and women. Clear cell was the most common RCC type, with about the same occurrence among men (*n* = 2159; 70.0%) and women (*n* = 1139; 72.5%). However, the distribution of RCC type did differ significantly between sexes (*P* < 0.001), with papillary RCC being more common among men (*n* = 553; 17.9%) than women (*n* = 178; 11.3%), and chromophobe RCC being more common among women (*n* = 176; 11.2%) than men (*n* = 257; 8.3%).

### Sex differences in health-related quality of life

Total and domain-specific univariate FKSI-14 and FKSI-19 scores are given in Table 2. Men had higher total and domain-specific scores than women, implying a generally higher HRQoL. All differences were statistically significant, except for the FKSI-19 domain Function/Well-being. Results from simple and multiple linear regression models (Table 3) show similar results. Men reported significantly higher HRQoL than women, except for the FKSI-14 domain Daily life function and well-being (multiple regression model) and the FKSI-19 domain Function/Well-being (simple and multiple regression models).

**Table 2:** Health‑related quality of life measured using the FKSI-14 and FKSI-19 instruments

FKSI version		Male	Female	*P*-value^a^	Missing values, *n* (%)
	Domain				
FKSI-14	*Physical and mental symptoms (0–45 points)*				
	Mean (SD)	29.2 (6.1)	27.9 (6.7)	**< 0.001**	34 (0.7)
	Median (IQR)	31 (26–34)	30 (24–33)		
	*Daily life function and well-being (0–20 points)*				
	Mean (SD)	13.8 (4.1)	13.4 (4.3)	**0.001**	47 (1.0)
	Median (IQR)	15 (12–17)	14 (11–16)		
	*Total (0–56 points)*				
	Mean (SD)	43.0 (9.1)	41.3 (9.8)	**< 0.001**	90 (1.9)
	Median (IQR)	45 (38–50)	43 (36–49)		
FKSI-19	*Disease-related symptoms – Physical (0–48 points)*				
	Mean (SD)	39.4 (6.4)	38.2 (7.1)	**< 0.001**	36 (0.8)
	Median (IQR)	41 (36–44)	40 (34–44)		
	*Disease-related symptoms – Emotional (0–4 points)*				
	Mean (SD)	2.75 (1.17)	2.49 (1.25)	**< 0.001**	50 (1.1)
	Median (IQR)	3 (2–4)	3 (2–4)		
	*Treatment side effects (0–12 points)*				
	Mean (SD)	11.0 (1.6)	10.8 (1.8)	**0.001**	82 (1.8)
	Median (IQR)	12 (11–12)	12 (10–12)		
	*Function/Well-being (0–12 points)*				
	Mean (SD)	8.4 (2.9)	8.3 (3.0)	0.314	51 (1.1)
	Median (IQR)	9 (7–11)	9 (6–10)		
	*Total (0–76 points)*				
	Mean (SD)	61.6 (10.1)	59.8 (10.9)	**< 0.001**	116 (2.5)
	Median (IQR)	64 (56–69)	62 (54–68)		

FKSI, functional assessment of cancer therapy – kidney symptom index; IQR, interquartile range; SD, standard deviation. Significant *P*-values are given in **bold**. ^a^*P*-values calculated using the Mann-Whitney test

**Table 3:** Results from simple and multiple linear regression models for the association between male sex (reference category: female sex) and health‑related quality of life measured using the FKSI-14 and FKSI-19 instruments

FKSI version				Male sex			
	Domain	Model	n (%)^a^	β	95% CI	*P*-value	R^2^ (%)
FKSI-14	Physical and mental symptoms (0–45 points)	Simple	4624 (99.3)	1.24	0.84, 1.64	**< 0.001**	0.9
		Multiple^b^	4317 (92.7)	0.95	0.56, 1.34	**< 0.001**	15.6
	Daily life function and well-being (0–20 points)	Simple	4611 (99.0)	0.44	0.18, 0.71	**< 0.001**	0.2
		Multiple^b^	4316 (92.7)	0.14	-0.11, 0.39	0.257	17.9
	Total (0–56 points)	Simple	4568 (98.1)	1.68	1.09, 2.28	**< 0.001**	0.7
		Multiple^b^	4282 (91.9)	1.10	0.54, 1.66	**< 0.001**	19.1
FKSI-19	Disease-related symptoms – Physical (0–48 points)	Simple	4622 (99.2)	1.16	0.73, 1.58	**< 0.001**	0.7
		Multiple^b^	4314 (92.6)	0.79	0.38, 1.20	**< 0.001**	14.6
	Disease-related symptoms – Emotional (0–4 points)	Simple	4608 (98.9)	0.26	0.18, 0.34	**< 0.001**	1.0
		Multiple^b^	4306 (92.4)	0.25	0.17, 0.33	**< 0.001**	8.8
	Treatment side effects (0–12 points)	Simple	4576 (98.2)	0.19	0.08, 0.30	**< 0.001**	0.3
		Multiple^b^	4284 (92.0)	0.12	0.01, 0.23	**0.022**	11.3
	Function/Well-being (0–12 points)	Simple	4607 (98.9)	0.10	-0.08, 0.29	0.262	0.03
		Multiple^b^	4313 (92.6)	-0.11	-0.27, 0.07	0.210	20.8
	Total (0–76 points)	Simple	4542 (97.5)	1.75	1.09, 2.42	**< 0.001**	0.6
		Multiple^b^	4256 (91.4)	1.08	0.46, 1.71	**< 0.001**	18.9

BMI, body mass index; CI, confidence interval; FKSI, functional assessment of cancer therapy – kidney symptom index; RCC, renal cell carcinoma. Significant *P*-values are given in **bold**. ^a^Number (%) of observations included in the model. ^b^Adjusted for age at surgery, education level (reference category: compulsory school), married/cohabiting/partner, employed, on sick leave, retired, BMI, time from RCC diagnosis to surgery, time from surgery to answering the questionnaire, currently on treatment for RCC, comorbidity index, tumour size, metastatic RCC, open surgery, T stage (reference category: T1), and RCC type (reference category: clear cell)

After adjusting for all included variables, men had on average 0.95 (95% CI 0.56–1.34) points higher HRQoL than women in terms of physical and mental symptoms according to the FKSI-14. According to the FKSI-19, the HRQoL among men was on average 0.79 (95% CI 0.38–1.20) points higher for physical disease-related symptoms, 0.25 (95% CI 0.17–0.33) points higher for emotional disease-related symptoms, and 0.12 (95% CI 0.01–0.23) points higher for treatment side effects. For the FKSI-14 and FKSI-19 total scores, which measure overall HRQoL, men had scores that were on average 1.10 (95% CI 0.54–1.66) and 1.08 (95% CI 0.46–1.71) points higher, respectively, than those of women.

### Non-response analysis

Compared with respondents, the 2489 patients that did not respond to the study questionnaire (Fig. 1) were younger, mean (SD) age 64.3 (13.0) years (*P* < 0.001), had a higher BMI, 28.0 (5.2) kg/m^2^ (*P* = 0.002), a longer time from RCC diagnosis to surgery, 104.5 (116.6) days (*P* = 0.020), and a lower T-stage, *n* = 1199 (48.6%) for T1a and *n* = 545 (22.1%) for T3–T4 (*P* = 0.035). There were no significant differences regarding sex (*P* = 0.752), tumour size (*P* = 0.179), metastatic status (*P* = 0.495), or RCC type (*P* = 0.230).

## Discussion

Using a large nation-wide cohort of surgically treated Swedish RCC patients, we found that men had significantly higher overall HRQoL than women, particularly regarding physical and emotional/mental symptoms and treatment-related side-effects, six months post-surgery. No significant sex differences were observed in daily life function and well-being. The results were similar between the FKSI-14 and FKSI-19 versions, indicating that they capture the same underlying HRQoL constructs.

### Results in perspective

Although the present study examined HRQoL at six months post-surgery and not at the time of diagnosis or during treatment, our findings align well with previous research indicating sex differences in psychological distress and HRQoL among RCC patients, with distress being more prevalent among women particularly at the time of diagnosis and during treatment. Bergerot et al. [32] found that female sex, younger age, non-clear cell histology, and disease recurrence were significantly associated with distress in a US-based cross-sectional study of RCC patients from an advocacy program. However, they did not account for socio-economic status or comorbidities, nor did they report time since diagnosis. Similarly, Ajaj et al. [18] reported that female sex and younger age were linked to higher distress at diagnosis and after surgery among non-metastatic RCC patients, even after accounting for demographic and clinical characteristics.

Some studies have suggested that age is a stronger predictor of distress than sex. Bahlburg et al. [33] found that younger age was the primary predictor of psychological distress after nephrectomy in cancer patients. In contrast, Draeger et al. [34] observed significant distress levels at diagnosis among many RCC patients, but found that distress levels were not significantly impacted by age, sex, treatment, or disease stage. Although an RCC diagnosis and treatment initially seem to increase distress, especially among women, Ajaj et al. [18] reported attenuated sex differences in distress over time, with no significant differences at 2–3 years post-diagnosis. This suggests that sex disparities in distress and subsequently HRQoL may decrease as patients adjust post-treatment. However, Beisland et al. [19] found that men reported higher HRQoL than women up to 14 years after diagnosis in a cohort of surgically treated RCC patients in Norway.

The reasons for these sex differences in HRQoL remain unclear. Although not addressed in this study, hypothetical explanations may include biological factors, such as hormonal influences, which may play a role, along with psychosocial differences in coping strategies and social support. Healthcare disparities, including differences in access to psychological support, may also contribute. Loneliness has been associated with lower HRQoL among older adults [35, 36], and women in our data were less likely than men to report being married, cohabiting, or having a partner. However, the observed higher HRQoL among men remained even after adjusting for relationship status in the regression models. Nevertheless, as our study design does not allow for causal interpretations, there is a clear need for future research to explore the causal mechanisms behind potential sex differences in HRQoL.

Differences in results between studies may be due to variations in study design, population characteristics, and assessment tools [37, 38]. Additionally, socioeconomic status and comorbidities, which may influence HRQoL, have not been consistently accounted for in previous research. Future studies should aim to clarify the relative contributions of these factors to better understand and address sex-based disparities in HRQoL in RCC patients.

### Kidney cancer compared to other types of cancer and the general population

Previous studies comparing HRQoL between RCC patients and those with other urological cancer diagnoses are limited, with mixed findings on distress and psychosocial well-being. While psychosocial distress is common in prostate, bladder, and kidney cancer patients [33], the extent of the differences between the cancers showed treatment and disease-specific independent predictors for high psychosocial distress after surgical treatment. Although Yang et al. [39] found no significant differences in psychological disorders between kidney and bladder cancer patients six months post-diagnosis, Pastore et al. [40] reported that patients undergoing radical nephrectomy reported higher anxiety scores than those undergoing radical cystectomy. Pastore et al. also found that females and patients with kidney tumours experienced higher levels of psychological distress than males and patients with bladder or prostate tumours, the latter independent of age and sex. Thus, treatment-related differences may contribute to variations in HRQoL between different malignancies.

While HRQoL generally improves post-surgery, the recovery trajectories may vary across domains and patient subgroups. Some studies suggest that reductions in HRQoL seen following surgery return to pre-operative levels over time [13, 41]. However, Kent et al. [42] found that physical HRQoL remained lower in RCC patients than in the general population more than three years after surgery, despite mental HRQoL being comparable. Conversely, other studies [16, 17] have indicate that overall HRQoL in RCC patients are comparable to population norms within one to four years after surgery. Nevertheless, despite improvements in HRQoL, concerns about cancer recurrence may persist for a long time [13]. Future research should explore long-term HRQoL determinants, including age, comorbidities, treatment type, and socioeconomic status, to identify patients at risk for prolonged impairment.

### Clinical implications

There are previous reports that women report worse HRQoL compared to men [43]. However, a recent study in the general Swedish population comprising 29,212 women and men aged 50–64 years, concluded that levels of physical and mental HRQoL measured using a validated Swedish version of the SF-12 questionnaire were similar between the sexes [44]. Nevertheless, it should be noted that men had higher physical HRQoL scores than women, although no formal statistical test comparing the two groups was performed. Our findings of significant sex differences in HRQoL among RCC patients may partly reflect the gender gap in HRQoL observed in the general population, rather than being disease specific, but still suggest a need for personalized supportive care strategies to reduce these differences. Healthcare providers should ensure that all patients feel heard and supported in making treatment decisions, e.g. through shared decision-making, while also recognizing the potential need for sex-specific communication strategies. Integrating psychosocial support into routine care is crucial for identifying and assisting patients in need of support as early as possible. Additionally, rehabilitation programs should include emotional and social well-being, alongside physical recovery, to improve overall patient well-being.

### Strengths and limitations

A major strength of our study was the large sample size and that we adjusted our analyses for both background characteristics and clinical variables that may influence patients’ HRQoL. Additionally, the data used in the study were sourced from the NSKCR, which provides a near-complete coverage of all surgically treated RCC patients in Sweden [45], increasing the generalizability of our results.

Among the limitations were the differences between responders and non-responders, with the non-responders being healthier than the responders, and the percentage of patients with metastatic disease being lower in our study (5%) than the overall level in the NSKCR (approximately 15%) [3], which affect the generalizability of the results. Another limitation was that it was not always possible to track the specific reason for why a patient did not obtain the questionnaire. Additional limitations include the cross-sectional design and lack of preoperative HRQoL data, meaning that we can only make statements about the level of HRQoL at specific time points and cannot address changes over time. Prior research suggests that sex differences in distress, which may impact quality of life, diminish after 2–3 years [18], but our study only captures HRQoL at six months post-surgery.

Moreover, using a kidney cancer-specific questionnaire limits the possibilities to make direct comparisons with HRQoL levels in the general population. A limitation of the FKSI-14/19 questionnaires is the lack of formally validated thresholds for defining clinically relevant differences between groups. When interpreting our findings, it is important to distinguish between statistical significance and clinical relevance. This is often done using the concept of minimally important difference (MID), which represents the smallest score difference likely to be meaningful to patients and clinicians. While a change of approximately 2–3 points has been suggested as a threshold for within-person change [46], a difference of around 1 point has been suggested as meaningful when evaluating between-group differences [47]. Finally, the use of different assessment methods addressing HRQoL in RCC patients, which is recognized as a general limitation to the field [38], makes comparisons between studies difficult.

## Conclusions

In this population-based study, HRQoL differed significantly between men and women six months after RCC surgery. Men reported higher overall HRQoL, and in particular in terms of physical and emotional/mental symptoms and side-effects from the treatment. To reduce sex differences in HRQoL among RCC patients, healthcare professionals should be aware of the risk of a lower HRQoL among females.

## Abbreviations

BMI: Body mass index
CI: Confidence interval
DLFWB: Daily life function and well-being
DRS-E: Disease-related symptoms – Emotional
DRS-P: Disease-related symptoms – Physical
FKSI: Functional assessment of cancer therapy – kidney symptom index
FWB: Function/well-being
HRQoL: Health-related quality of life
IQR: Interquartile range
MID: Minimally important difference
NSKCR: National swedish kidney cancer register
PMS: Physical and mental symptoms
PROM: Patient reported outcome measures
RCC: Renal cell carcinoma
QoL: Quality of life
SD: Standard deviation
STROBE: Strengthening the reporting of observational studies in epidemiology
TNM: Tumour-node-metastasis
TSE: Treatment side effects
WHO: World Health Organization
